# Piezoelectric conductive electrospun nanocomposite PCL/Polyaniline/Barium Titanate scaffold for tissue engineering applications

**DOI:** 10.1038/s41598-022-25332-w

**Published:** 2022-12-02

**Authors:** Naeemeh Peidavosi, Mahmoud Azami, Nima Beheshtizadeh, Ahmad Ramazani Saadatabadi

**Affiliations:** 1 Department of Biomedical Engineering, Science and Research Branch, Islamic Azad University, Tehran, Iran; 2 Department of Tissue Engineering, School of Advanced Technologies in Medicine, Tehran University of Medical Sciences, Tehran, Iran; 3 Department of Chemical and Petroleum Engineering, Sharif University of Technology, Tehran, Iran

**Keywords:** Translational research, Biomedical engineering, Biomaterials

## Abstract

Recent trends in tissue engineering technology have switched to electrical potentials generated through bioactive scaffolds regarding their appropriate effects on cell behaviors. Preparing a piezo-electrical stimuli scaffold with high electrical conductivity for bone and cartilage tissue regeneration is the ultimate goal of the present study. Here, Barium Titanate nanoparticles (BaTiO_3_ NPs) were used as piezoelectric material and highly conductive binary doped Polyaniline nanoparticles (PANI NPs) were synthesized by oxidative polymerization. Polycaprolactone (PCL) was applied as carrier substrate polymer and conductive spun nanofibrous scaffolds of PCL/PANI composites were prepared in two different amounts of PANI (3 and 5 wt.%). The conductivity of PCL/PANI nanofibers has been analyzed by standard four probes test. Based on the obtained results, the PCL/PANI5 (with 5 wt.% PANI) was selected due to the superior electrical conductivity of 8.06 × 10^–4^ s cm ^- 1^. Moreover, the piezoelectric nanofibrous scaffolds of PCL/BT composite were electrospun in three different amounts of BT (20, 30, and 40 wt.%). To investigate the synergic effect of conductive PANI and piezoelectric BT, ternary nanocomposite scaffolds of PCL/PANI/BT were prepared using the dual jet electrospinning technique. The piezoelectric properties have been analyzed by determining the produced voltage. The morphological assessment, contact angle, mechanical test, and MTT assay have been conducted to evaluate other properties including biocompatibility of nanofibrous scaffolds. The PCL/PANI5/BT40 composite resulted in an unprecedented voltage of 1.9 Volt. SEM results confirm that BT NPs have been distributed and embedded inside PCL fibers quite appropriately. Also, the chosen scaffolds were homogeneously intertwined and possessed an average fiber diameter of 288 ± 180 nm, and a contact angle of 92 ± 7°, making it a desirable surface for cell attachment and protein interactions. Moreover, Young’s modulus, ultimate tensile stress, and elongation were obtained as 11 ± 1 MPa, 5 ± 0.6 MPa, and 109 ± 15% respectively. Obtained results assert the novel potential of piezo-electrical stimuli conductive nanocomposite scaffold for tissue engineering applications.

## Introduction

The specific aim of tissue engineering (TE) is to assemble cells with biomaterials (scaffolds) and growth factors to repair, regenerate, improve or replace damaged or lost tissues or whole organs^1^. Regarding promising development in biomaterial science and tissue engineering, applications of new biomaterials with smart, precisely controllable bio-functions have been expanded^2^. Smart materials are able to respond to stimuli by adjusting their functions or properties due to any specific external or even internal stimulus^3^.

Piezoelectric materials, with the ability to transform mechanical energy to electrical energy, are considered smart materials^4,5^. From numerous studies, it has been proven that bioactive, smart scaffolds, especially electrical stimuli, play significant roles in the regeneration and repairing of defected tissues^6,7^. Scaffolds in addition to providing a supportive substrate for cells attachment and proliferation must be able to mimic the natural tissue extracellular matrix (ECM)^8^. Some tissues such as bone, tendon, and cartilage that contain large amounts of collagen, are inherently piezoelectric^9^, which means they are able to render electrical signals in response to applying strain and deformation. This phenomenon has raised an active potential in the tissue engineering field. Therefore, an optimized piezoelectric and conductive scaffold, which is able to produce appropriate bioelectrical signals like natural ECM, and transmit the generated signals all over the scaffold is desirable^10^.

Lead-free, ferroelectric Barium Titanate (BT) is classified as bio piezoelectric materials with a high coefficient of piezoelectricity of 191 pc/N^5^. It has been proven to be a successful candidate for a wide range of medical applications due to its improved biocompatibility even at high concentrations^5^. Multiple studies have reported its supportive role in cell attachment and proliferation in bone scaffolds, due to its TiO_2_ component, moreover, BT promotes scaffold's mechanical properties^11^.

To induce conducting property and have a synergic result with piezoelectricity, Polyaniline (PANI) was chosen among conducting polymers due to its unique properties like easy polymerization, variable doping stage, and conducting stability in spite of its poor solubility, processability, and also rigid nature^12^. Since PANI is rather infusible in the most common solvents, its biomedical application has been severely restricted^13^. Many efforts have been done to reduce these drawbacks by replacing other alternative dopant agents and blending them with proper polymers.

Polycaprolactone (PCL) as a biodegradable, biocompatible, and FDA-approved polymer has been used previously as the whole or part of several scaffolds in tissue engineering^14,15^. PCL offers proper physical and mechanical properties for tissue engineering scaffolds, such as appropriate solvability in common solvents, slow degradation rate with non-toxic products of degradation, and remarkable ability to blend with different polymers^16^.

As far as we know, numerous studies have been conducted on bioactive tissue engineering scaffolds; however, a limited number of studies investigated the electrical stimuli scaffolds with synchronous conductivity^17,18^. The ultimate goal of the present study was to design and prepare a self-electrical stimuli tissue engineering electrospun scaffold with high conductivity and modifiable output voltage to regenerate tissues like bone and cartilage. Firstly, PANI NPs were synthesized by oxidative polymerization. Then varying amounts of BT and PANI NPS were blended in PCL substrate, dual and ternary nanocomposites with high porosity structure were synthesized by electrospinning method. Eventually, the chemical, physical, morphological, mechanical, and biocompatibility properties of nanocomposite mats were compared and investigated. The given results emphasize the feasibility of the synthesized scaffold for bone and cartilage tissue regeneration.

## Materials and methods

### Materials

PCL with a molecular weight of 80,000 g/mol and (1S)-(+)-10-Camphorsulfonic acid (CSA), were purchased from Sigma-Aldrich (Inc. St. Louis, MO. USA). Aniline (99.0%, monomer), Ammonium Peroxydisulfate (APS), N,N-dimethyl formamide (DMF), Hydrochloric acid (HCL), Barium Titanate (BaTiO_3_), Chloroform (CHF), methanol, and Acetone were purchased from Merk (Darmstast, Germany). Fetal bovine serum (FBS), Phosphate-buffered saline (PBS), 3-(4,5-dimethylthiazol-2-yl)-2,5-diphenyltetrazolium bromide (MTT), RPMI 1640 (culture medium), trypsin–EDTA, Penicillin–Streptomycin, and Isopropanol were supplied from Sigma–Aldrich. MG-63 cell line, were obtained from Pasteur cell bank of Iran. Aniline was purified by distillation in vacuum before use.

### Synthesis and characterization of PANI NPs

PANI in emeraldine salt (ES) form was synthesized according to MacDiarmid and Epstein method^19^. For this aim, 1 ml of Aniline monomer was added in 100 ml 1 M HCl into a three-necked round bottom flask and stirred for 1 h under argon atmosphere at 0–5 °C within an ice bath. The oxidant APS (1.24 g) was dissolved in 20 ml (1 M) HCl. APS solution was dropped into HCL/Aniline solution by a syringe pump in a quite slow mode (to obtain uniform nano-sized particles) and was continuously stirred for 6 h. in an argon atmosphere at 0–5 °C. A dark green solution was kept for 24 h at 0–5 °C to complete polymerization. In order to eliminate HCl and unwanted Aniline oligomers, the polymer product was washed with Acetone and centrifuged at 10,000 rpm for 15 min. This was repeated until the washing liquid collected on the falcon was colorless. The obtained paste was dried under vacuum for 24 h.

The chemical structures and formation of chemical groups of the synthesized PANI were evaluated by FTIR spectroscopy over the range of 4000–400 cm^-1^ and recorded on a transform infrared spectrometer (AVATAR-Thermo, USA). The crystalline structure was identified by using x-ray diffraction (XRD, PW1730, PHILIPS), the morphology and size of the PANI nanoparticles were studied using field emission scanning electron microscopy (FE-SEM, MIRA-III, TESCAN).

### Fabrication of nanofibrous hybrid scaffolds

Nanofibrous scaffolds were prepared using the electrospinning method. For this aim, PCL solution with the concentration of 12% w/v was prepared by dissolving a certain amount of PCL in 5 ml of mixed solvents of CHF/methanol with the volume ratio of 1:1 and mixed under a magnetic stirrer for 3 h at room temperature. The PCL solution was added to a 5 ml syringe equipped with an 18 G needle and electrospinning was done applying 20 kV high voltage, 13 cm distance between collector and the needle, and 1 mL/h solution rate.

To prepare piezoelectric nanofibers with the composition of PCL/BT, 20, 30, and 40 wt.% BT NPs were dispersed in 2.5 mL methanol (Table 1) using a magnetic stirrer for 20 min followed by sonication for 10 min. The process was repeated until obtaining a homogeneous white suspension. Then the suspension was added gradually to the PCL solution (12% w/v in 2.5 ml CHF) and electrospun with the same condition as described earlier for the PCL solution.

**Table 1:** Composition of electrospun PCL, PCL/PANI and PCL/BT.

Samples	PCL (wt./wt.%)	PANI (wt./wt.%)	BT (wt./wt.%)
PCL	100	0	0
PCL/PANI3	97	3	0
PCL/PANI5	95	5	0
PCL/BT20	80	0	20
PCL/BT30	70	0	30
PCL/BT40	60	0	40

Electroconductive nanofibers of PCL/PANI were prepared by dispersing of 3 and 5 wt.% PANI NPs and Camphor Sulfonic acid (CSA) (a compatible dopant agent) in 2.5 ml DMF (Table 1), with the ratio of 1:1.24 for PANI: CSA. The mixture was sonicated for 10 min and then stirred for 20 min. The process was repeated four times and finally stirred for 24 h. The PANI/CSA solution was mixed gradually with the PCL solution (12% w/v in 2.5 ml CHF) and was electrospun in the same condition as mentioned above.

The PANI NPs were synthesized in conductive emeraldine salt form, which was previously doped by HCl acid during polymerization (as described in Sect. 2.2). A proper amount of CSA is employed to enhance the conductivity of PANI. Binary doping led to a significant increase in electrical conductivity. Furthermore, CSA can act as a surfactant in the aqueous solutions to improve the PANI’s processability, solvability, and stability^20,21^. The resulting solution was in deep green color.

To prepare a piezoelectric conductive nanocomposite scaffold composed of PCL/PANI5/BT20, the dual jet electrospinning technique was employed (Table 2). The electroconductive PCL/PANI5 and piezoelectric PCL/BT20 solutions were poured into two different 5 ml syringes equipped with an 18 G needle. A syringe with PCL/PANI solution was placed at one side (Jet 1), and syringe containing PCL/BT was placed in the opposite side (Jet 2). The electrospinning conditions between the Jet 1 and Jet 2 were kept the same as described above for PCL electrospinning, including 20 kV high voltage, 13 cm distance, and 1 ml/h flow rate. Same process was repeated for PCL/PANI5 and PCL/BT30, and also PCL/PANI5 and PCL/BT40 solutions, to obtain ternary nanocomposite scaffolds of PCL/PANI5/BT30 and PCL/PANI5/BT40.

**Table 2:** Different combination of electrospun PCL/PANI5 and PCL/BT in dual jet electrospinning.

Ternary nanocomposite Scaffolds	Jet1	Jet2
PCL/PANI5/BT20	PCL/PANI5	PCL/BT20
PCL/PANI5/BT30	PCL/PANI5	PCL/BT30
PCL/PANI5/BT40	PCL/PANI5	PCL/BT40

## Characterization of nanofibrous scaffolds

### Physicochemical characterization

*FTIR* (Fourier-transform infrared spectroscopy): To investigate the chemical bonds and functional groups within the PCL, PCL/PANI5, and PCL/BT40 nanofibrous scaffolds, FTIR (AVATAR-Thermo, USA) was done at transmittance mode. Fibrous mats were shredded mixed with KBr and palletized, then the spectra were recorded over a range of 4000–400 cm^−1^.

*SEM* (Scanning electron microscope): Morphology of nanofibers and distribution of BT NPs were investigated using SEM (PHILIPS XL30). To obtain the average diameter and its standard deviation of nanofibers, approximately 60 different fibers of each image were randomly selected and calculated using Image-J software.

*Water contact angle:* Wettability of prepared nanocomposite fibrous scaffolds and also the effect of adding nanoparticles on the scaffolds were studied by the water contact angle method. For this aim, a droplet of deionized water (4 µL) was added to the surface of (2 × 2 cm^2^) specimens and 3 series of images were taken. The average degree was calculated and reported as water contact angle.

*Mechanical characterization:* Mechanical characterization of the prepared samples was evaluated under compression test using a uniaxial tensile testing machine (Zwick Roell, Proline tensile tester). To do that, samples were cut into (30 × 5mm^2^) strips and held between two vertical clamps. All specimens were pulled at 10 mm min^−1^ constant speed until breakpoint. To ensure the correctness of the test each sample was tested 3 times with the same method and identical conditions. The stress–strain curves were plotted; the ultimate tensile stress, strain, and Young’s modulus were calculated.

*Piezoelectric properties and electrical conductivity:* The resulting voltages of piezoelectric nanocomposite scaffolds (PCL/BT and PCL/PANI/BT) were evaluated by a piezo tester device. Measurement of the output voltage is the main parameter to determine piezoelectric properties. Constant cyclic forces were applied to the samples and the electric output was recorded using a digital oscilloscope (ROHDE& SCHWARZ, HMO_3522, Germany). Each nanocomposite mat was cut into (2 × 1 cm^2^) strips and was subjected to a compressive cyclic force of 2.66 N and 5 Hz. To ensure the accuracy of the test, each sample was tested 3 times with the same method and identical conditions.

To analyze the electrical conductivity of PCL/PANI nanofibers, a standard four-probes method was used. Four probes with the same distance were located in one direction. The specified current (1.4 mA) is fed to the first and last probes and voltage is measured from the two middle probes. The electrical conductivity is calculated by knowing the thickness of the nanofibrous scaffolds and using the following Eq. (1):

1 $$\sigma =\frac{ln2}{\pi t}\left(\frac{I}{V}\right)$$

where σ, t, I and V are electrical conductivity (S.cm^-1^), thickness (cm), current (A), and voltage (v), respectively^22^.

### Biocompatibility and cell viability evaluation

In order to analyze the viability and proliferation of cells grown on nanofibrous scaffolds, the MTT assay was used. For this aim, MG-63 cells were cultured in RPMI 1640 (10% FBS, 1% penicillin–streptomycin). The culture medium was replaced every 3 or 4 days. All scaffolds were sterilized with ethanol (70% v/v) and UV radiation. 2 × 10^4^ cells in 100 µL culture medium were seeded on the prepared scaffolds in a 12-well culture plate. After 1 and 3 days, the culture medium was replaced with 400 µL MTT solution (0.5 mg/ml) and incubated for 4 h. Then, the medium was replaced with isopropanol and shook for 15 min to dissolve the formazan crystal. Finally, viability of the cultured cells was evaluated using the microplate reader (SASA FAX 2100, USA) at 570 nm according to the following Eq. (2).

2 $$\text{Cell viability }\left(\%\right)=\frac{\text{Absorbance of cells cultured with scaffold sample}}{\text{Absarbance of control cells}}$$

### Statistical analysis

All data were analyzed using paired comparison plot app by Origin Pro-2021. Data are presented as mean ± SD. * Indicated for P < 0.05, ** indicated for P < 0.01, *** indicated for P < 0.001. P value < 0.05 was considered as significant.

## Results and discussion

### PANI NPs characterization

**Figure 1:**
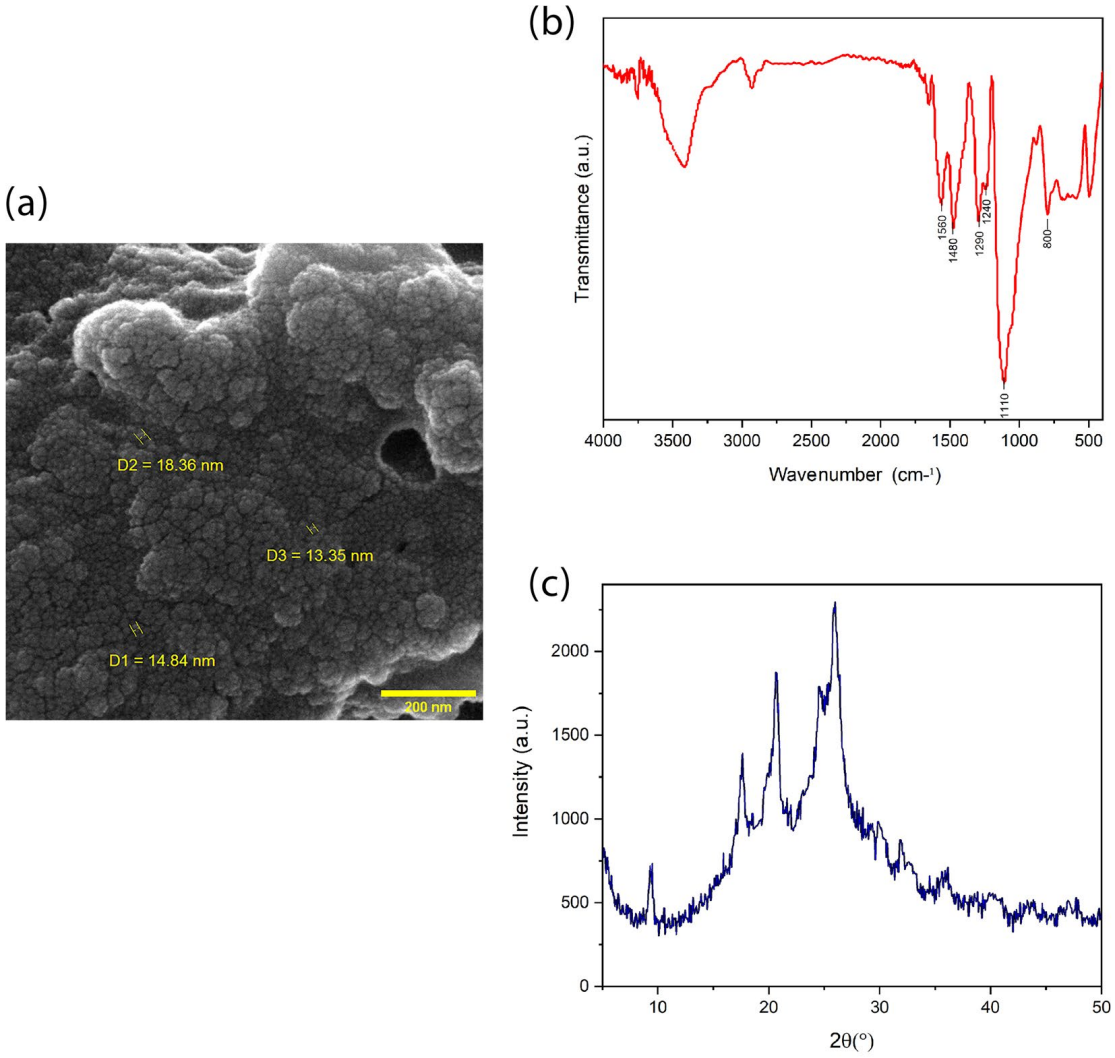
Analysis of synthesized PANI nanoparticles: (**a**) FE-SEM micrograph of synthesized PANI nanoparticles, which represents the spherical uniform distribution with an average diameter of 15 ± 2 nm, (**b**) FTIR spectra shows that the band near 1560 cm^−1^ and 1480 cm^−1^ related to C=C stretching deformation of Benzenoid and Quinoid rings, while the band absorbed at 1110 cm^−1^ pointed to the aromatic C–H in plane bonding. Also, the C–N stretching of the secondary amine of the PANI backbone is characterized by the peak at 1290 cm^−1^. (**c**) XRD pattern shows that all four major characteristic peaks with 2θ values are represented at 25.85°, 20.65°, 17.65°, and 9.5°. The sharp and well-defined peaks in the X-ray pattern indicated formation of a semi-crystalline structure of PANI NPs due to the Benzenoid and Quinoid functional groups.

The FE-SEM micrograph of synthesized PANI NPs demonstrated the shape and size of the NPs. As shown in Fig. 1a, the spherical uniform distribution with an average diameter of 15 ± 2 nm has been observed. The chemical structures of PANI NPs were performed using FTIR. Figure 1b, represent the FTIR spectra in the 4000–400 cm^−1^ range. The band near 1560 cm^−1^ and 1480 cm^−1^ related to C=C stretching deformation of Benzenoid and Quinoid rings. The band absorbed at 1110 cm^−1^ pointed to the aromatic C–H in plane bonding. The C–N stretching of the secondary amine of the PANI backbone is characterized by the peak at 1290 cm^−1^^23^.The band around 800 cm^−1^ is due to the vibration of C–H in the 1,4-disubstituted Benzene ring^24^. Also, the peak at 1240 cm^−1^ corresponded to emeraldine salt (ES) conducting form of PANI^22,23^.

The X-ray diffraction pattern of PANI NPs is shown in Fig. 1c. All four major characteristic peaks with 2θ values are represented at 25.85°, 20.65°, 17.65°, and 9.5°. The sharp, well-defined peaks in the X-ray pattern indicated formation of a semi-crystalline structure of PANI NPs due to the Benzenoid and Quinoid functional groups. Overall, the results of FTIR and XRD analyses confirmed successful formation of conductive PANI in this study.

### Characterization of nanofibrous scaffolds

#### FTIR

**Figure 2:**
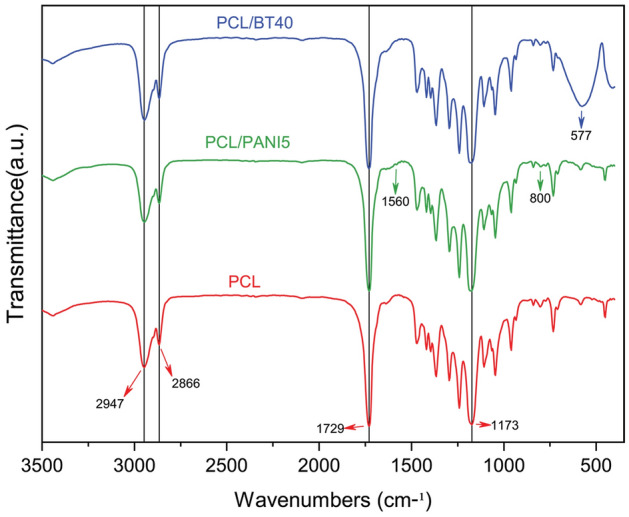
FTIR spectra of PCL, PCL/PANI5, and PCL/BT40 nanofibrous scaffolds. Studying the FTIR spectra of composite nanofibers reveals the presence of BT and PANI NPs within the fibrous scaffolds.

The FTIR spectra of PCL/PANI5 and PCL/BT40 were obtained and compared with pure PCL spectra between the wave numbers from 4000 to 400 cm^−1^ (Fig. 2). In the PCL spectra, all of the characteristic peaks were clearly identified. Peaks at 2947 cm^−1^ and 2866 cm^−1^ were attributed to the asymmetric and symmetric stretching vibration of CH2 groups, respectively. Furthermore, the strong absorptions at 1729 cm^−1^ and 1173 cm^−1^ are corresponding to (C=O) and (C–O) vibration, respectively. The weak absorptions at 1560 cm^−1^ (Quinoid rings) and 800 cm^−1^ (Benzenoid rings) on PCL/PANI5 spectra were assigned to the presence of PANI. Also, the strong absorption band at 577 cm^−1^, on PCL/BT40 spectra confirm the stretching (Ti–O) of BT NPs. Studying the FTIR spectra of composite nanofibers reveals the presence of BT and PANI NPs within the fibrous scaffolds.

#### Morphological observation

**Figure 3:**
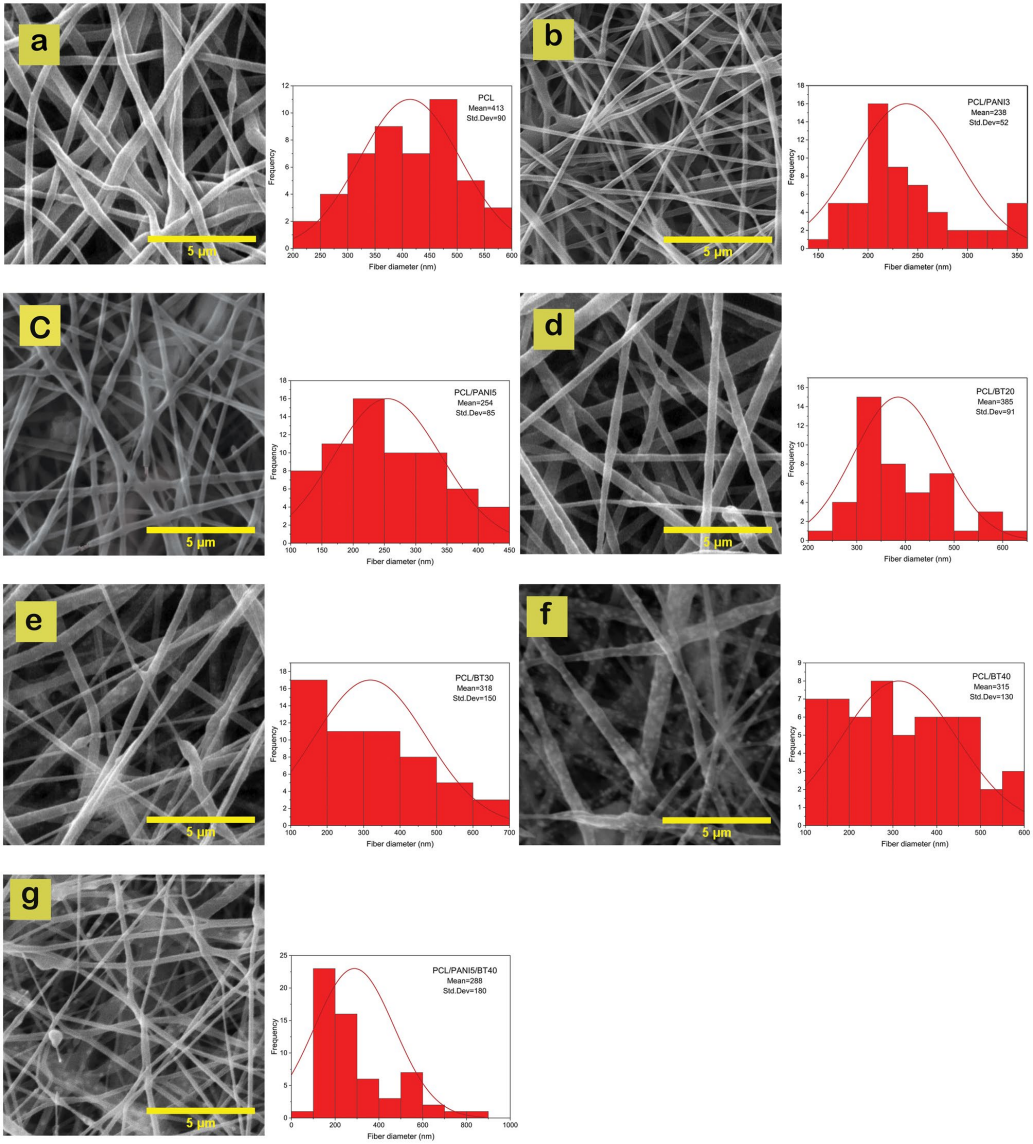
SEM micrograph of prepared scaffolds: (**a**) PCL (413 ± 90 nm average diameter), (**b**) PCL/PANI3 (238 ± 52 nm average diameter), (**c**) PCL/PANI5 (254 ± 85 nm average diameter), (**d**) PCL/BT20 (385 ± 91 nm average diameter), (**e**) PCL/BT30 (318 ± 150 nm average diameter), (**f**) PCL/BT40 (315 ± 130 nm average diameter), and (**g**) PCL/PANI5/BT40 (288 ± 180 nm average diameter) with respective fiber diameter distribution histograms. Results showed that in ternary nanocomposite PCL/PANI5/BT40, PCL nanofibers containing the PANI and BT NPs were homogeneously intertwined.

The SEM images demonstrate the morphology of nanocomposite fibrous scaffolds, as illustrated in Fig. 3. PCL nanofibers were observed to be smooth, uniform, and continuous without any beads or deformities. Fiber diameters were optimized by using a mixed solvent system of Chloroform and Methanol (1:1). The addition of Methanol (a high dielectric solvent) in an equal ratio resulted in to increase in the electrical conductivity of the PCL solution and reduction of nanofibers diameter^25^. The average diameters were calculated to be 413 ± 90 nm (Fig. 3a).

Viscosity and conductivity of the polymeric solution in electrospinning are two primary factors affecting the diameter of nanofibers^26,27^. It was shown that the addition of conductive PANI NPs increased the dielectric constant and thereupon the conductivity of the PCL solution^28^. The tension caused by the electric charges led to a decrease in the average diameter of the obtained PCL/PANI nanofibers^28^. For the samples with 3 and 5 wt.% PANI, fiber diameter was 238 ± 52 and 254 ± 85 nm, respectively (Fig. 3b,c). As presented in Fig. 3d–f, BT NPs added into the PCL solution, are uniformly distributed within the polymeric matrix and embedded inside the fibers and no rough lumps or beads were witnessed. However, a slight decrease in fiber diameters has been observed with increasing the percentage of BT. The average diameters of the PCL/BT nanofibers with 20, 30, and 40 wt.% BT were calculated to 385 ± 91,318 ± 150, and 315 ± 130 nm, respectively. This is due to the possible interaction between BT particles with electric fields formed during electrospinning^29^. High external electric voltage (20 kV) during electrospinning, causes the electrical dipoles inside BT particles to become parallel to the external electrical field and polarized^29^. The polarization increased the conductivity of the PCL blend solution resulting in the reduction of fiber diameters^30^. As shown in Fig. 3g, in ternary nanocomposite PCL/PANI5/BT40, PCL nanofibers containing the PANI and BT NPs were homogeneously intertwined. The average fiber diameter was calculated to 288 ± 180 nm.

#### Water contact angle

**Figure 4:**
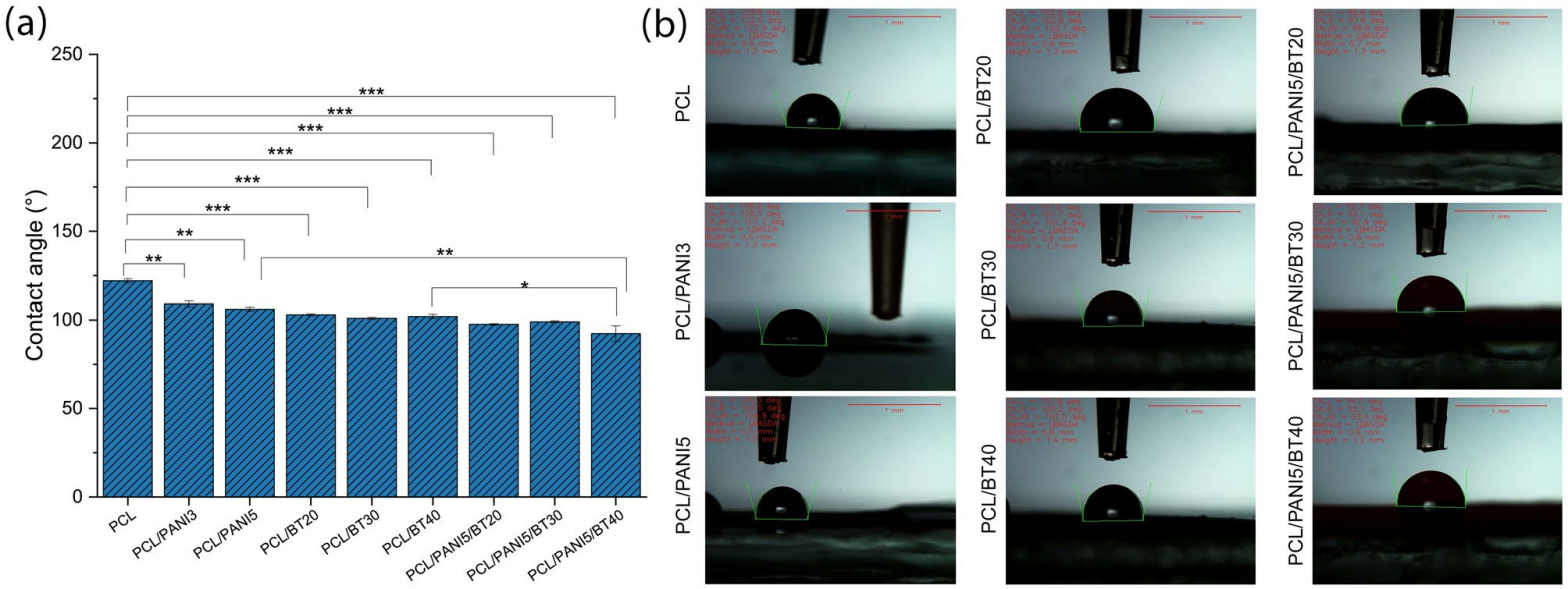
Water contact angle of nanofibrous scaffolds: (**a**) diagram, and (**b**) test images. The PCL offers hydrophobic nature, where its contact angle was measured to be 122 ± 2°. The addition of 3 and 5 wt.% PANI NPs resulted in approximately 10% reduction of the water contact angle to be 109 ± 3° and 106 ± 2°, respectively. Moreover, the addition of 20, 30, and 40 wt.% BT NPs led to a decrease in water contact angle of PCL matrix to 103 ± 1°, 101 ± 1°, and 102 ± 2°, respectively. In ternary nanocomposite of PCL/PANI/BT significant reduction of water contact angle (92 ± 7°) was observed, which made a desirable surface for cell attachment and protein interactions.

The surface properties and hydrophilicity of nanofiber scaffolds were analyzed by water contact angle test. The PCL offers hydrophobic nature, where its contact angle was measured to be 122 ± 2°. Hydrophobic nature is considered as one of the few disadvantages of PCL nanofibers that reduces the cell attachment tendency. To address this issue, multiple efforts have been done to modify PCL via surface treatment methods^31^. The contact angle of bulk PANI film was calculated to be 48.5 ± 0.8^25^, therefore dispersion of PANI in PCL matrix reduced the water contact angle of nanofiber composites. As shown in Fig. 4a, the addition of 3 and 5 wt.% PANI NPs resulted in approximately 10% reduction of the water contact angle to be 109 ± 3° and 106 ± 2°, respectively. It was shown that the addition of 20, 30, and 40 wt.% BT NPs led to a decrease in water contact angle of PCL matrix to 103 ± 1°, 101 ± 1°, and 102 ± 2°, respectively. It seems this is because of the hydrophilic nature of Titanate (TiO_2_) in BT. Consequently, in ternary nanocomposite of PCL/PANI/BT significant reduction of water contact angle was observed. Figure 4b depicts the water contact angle tests of prepared samples. In piezo-electrical stimuli conductive scaffold PCL/PANI5/BT40, the resulted contact angle was calculated to 92 ± 7°, which made a desirable surface for cell attachment and protein interactions.

#### Mechanical properties

**Figure 5:**
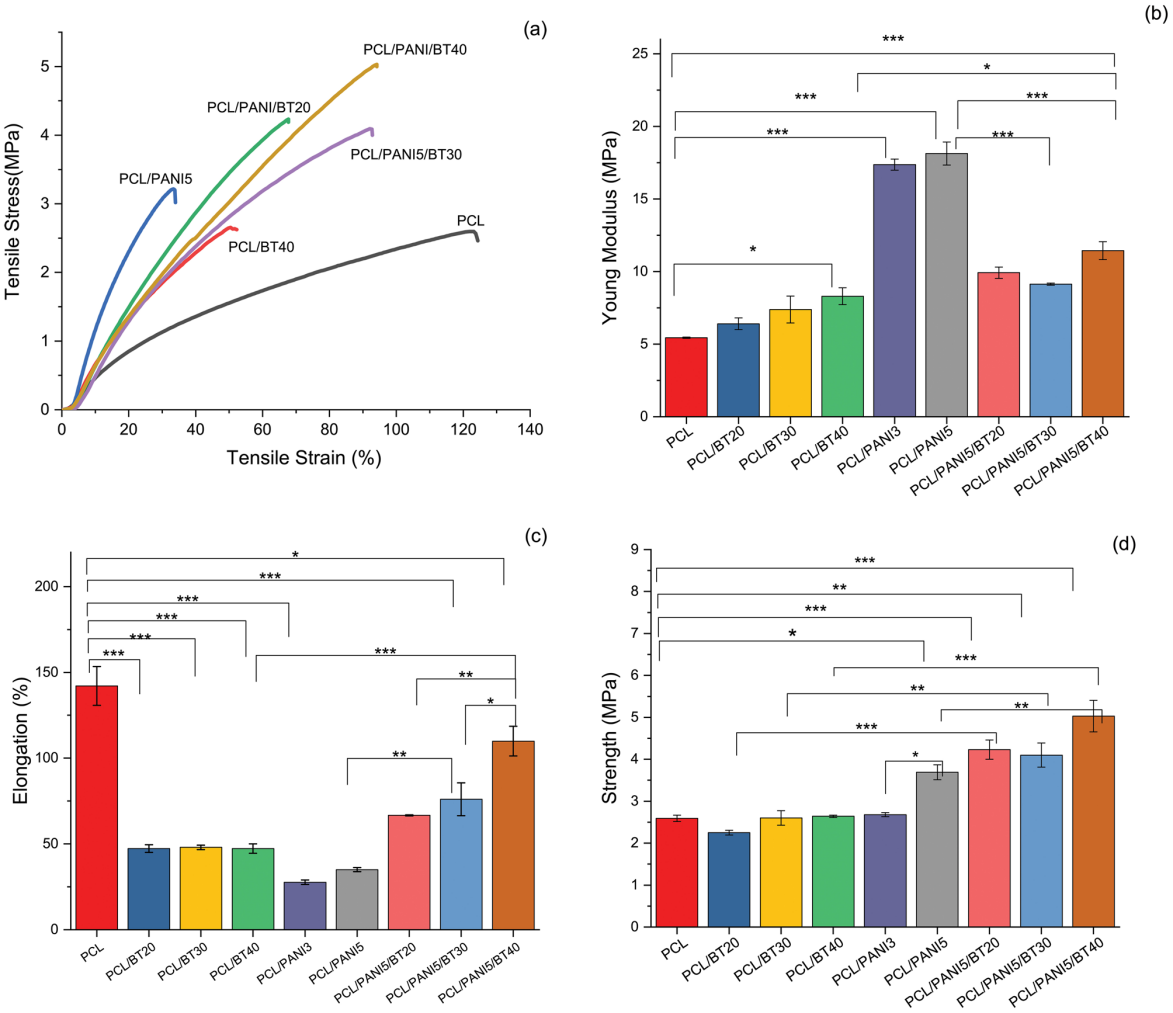
Mechanical properties of nanofibrous scaffolds: (**a**) stress–strain curves (**b**) Young’s modulus (**c**) Elongation at break, and (**d**) Tensile strength. Pure PCL nanofibrous scaffold has poor stiffness and UTS, but high elongation. The addition of PANI nanoparticles to the PCL, nanofibers gained stiffness, but gradually lost their elasticity, due to the PANI’s naturally hard and brittle properties. Adding BT NPs cause to increase in the Young’s modulus and decrease in elongation. Comparing ternary nanocomposite sample with others, stiffness has improved, and elasticity has set in a desirable amount for various tissues application.

Mechanical properties are one of the principal parameters in the performance of a bone and cartilage tissue engineering scaffold. The scaffolds must be strong enough to withstand the tensile and compressive loads during the host tissue regeneration^22^. Mechanical indices of prepared scaffolds were calculated from obtained stress–strain curves. The changes in the Young’s modulus and ultimate tensile stress (strength, MPa) and strain (elongation %) can be observed clearly in Fig. 5. Pure PCL nanofibers offered young’s modulus of 5.4 ± 0.08 MPa, ultimate tensile strain and stress of 142 ± 2% and 2.6 ± 0.1 MPa. It is concluded that pure PCL nanofibrous scaffold has poor stiffness and UTS, but high elongation.

Young’s modulus and ultimate stress of the PCL/PANI scaffolds were enhanced with increasing in PANI content to (17.4 ± 0.6, 2.7 ± 0.8) MPa and (18.1 ± 1, 3.7 ± 0.3) MPa for PCL/PANI with 3 and 5 wt.% PANI, respectively. However, tensile elongation of these samples reduced to 27 ± 2, 35 ± 2% respectively. This means that nanofibers gained stiffness, but gradually lost their elasticity, due to the PANI’s naturally hard and brittle properties. Inherently PANI has poor processability and this is one of the main disadvantages that limits PANI’s applications in biomedical fields^32^. As shown in a previous study^33^ combining PANI with elastic and ductile polymers enhances their mechanical properties.

In a similar way, adding BT NPs cause to increase in the Young’s modulus to 6.4 ± 0.7, 7.4 ± 1, and 8.3 ± 1 MPa for 20, 30, and 40 wt.% BT NPs, respectively. Meanwhile, it reduced the elongation to about 47%. This is due to the BT ceramic nature. In some studies, BT NPs were incorporated as second phase reinforcement to promote tensile strength. In the ternary nanocomposite PCL/PANI5/BT40, Young’s modulus and ultimate tensile stress raised to 11 ± 1 and 5 ± 0.6 MPa, and ultimate tensile strain (elongation %) dropped slightly to 109 ± 15%. Comparing ternary nanocomposite sample with binary nanocomposite fibers (PCL/PANI and PCL/BT), and pure PCL according to the Fig. 5a–d, stiffness has improved, and elasticity has set in a desirable amount for different tissues^34^.

#### Electrical conductivity

The electrical conductivity of nanofibrous scaffolds was analyzed using four probe devices. PCL nanofibers did not show any measurable conductivity, while by adding PANI NPs to the PCL substrate, a notable conductivity was experienced (Table 3). In the platform of PCL/PANI3, the conductivity was calculated to be 1.3 × 10^–4^ s cm^−1^. As expected, by increasing in PANI content, increase in conductivity was observed and reached to a considerable amount of 8 × 10^–4^ s cm^−1^ in PCL/PANI5 nanofiber. Due to a better conductivity result, sample of PCL/PANI5 was selected as an appropriate candidate for ternary nanocomposite scaffold PCL/PANI/BT.

**Table 3:** Electrical conductivity of nanofibers with 3 and 5 wt.% PANI.

Samples	PCL	PCL/PANI3	PCL/PANI5
Conductivity (S cm^−1^)	0	1.3 × 10^–4^	8.06 × 10^–4^

#### Piezoelectric properties

**Figure 6:**
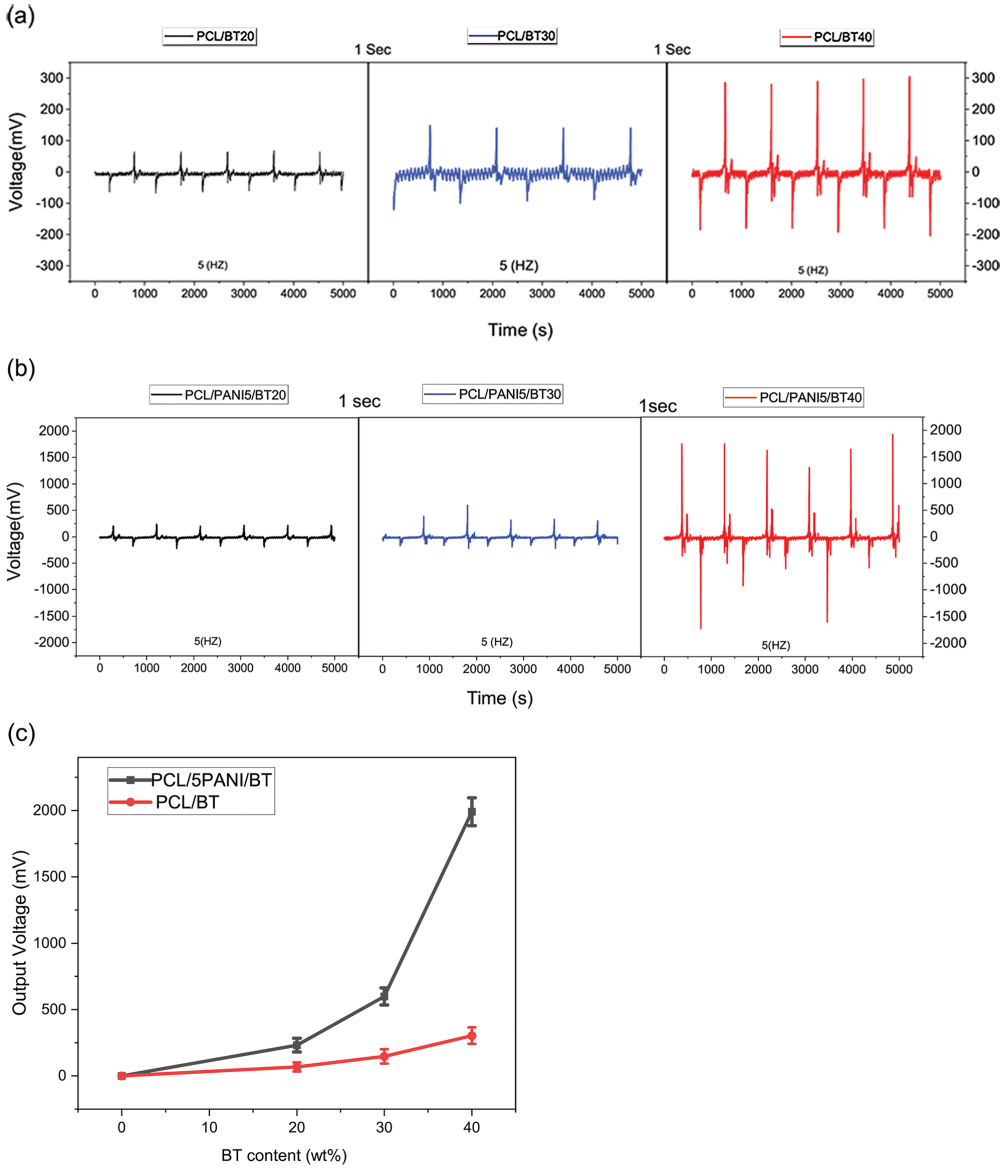
The recorded piezo-voltage of nanocomposite fibers. (**a**) BT content scaffolds; PCL/BT20, PCL/BT30, and PCL/BT40, (**b**) BT and PANI content scaffolds; PCL/PANI5/BT20, PCL/PANI5/BT30 and PCL/PANI5/BT40 (**c**) output voltage of PCL/BT versus PCL/PANI5/BT (20, 30, and 40 wt.% BT), comparison of piezo-voltage of containing PANI and non-containing PANI scaffolds. PANI NPs had a synergic effect on piezoelectricity and enhanced the output voltage of nanocomposites. Conductive PANI NPs assisted to decrease the internal resistance of ternary nanocomposite by introducing conductive paths.

The resulting voltage of piezoelectric nanocomposite scaffolds containing diverse amounts of BT NPs (20, 30, and 40 wt.%) and 5 wt.% PANI is illustrated in Fig. 6. The measured voltage is the main parameter for determining piezoelectric properties. The voltage was obtained by applying constant and cyclic force (2.66 N, 5 Hz), and the electric output was measured on an oscilloscope. As shown in Fig. 6, the voltage value is dependent on ferroelectric BT NPs content. Voltage in the sample with 20 wt.% BT is reported as 50 ± 6 mV, while for 30 and 40 wt.% the voltage values showed a significant increase to 148 ± 24 and 304 ± 12 mV, respectively (Table 4).

**Table 4:** Output voltage of nanocomposites with 20, 30, and 40 wt.% BT and Output voltage of ternary nanocomposites with 20, 30, and 40 wt.% BT and 5 wt.% PANI.

Samples	PCL/BT20	PCL/BT30	PCL/BT40	PCL/PANI5/BT20	PCL/PANI5/BT30	PCL/PANI5/BT40
Voltage (mv)	50 ± 6	148 ± 24	304 ± 12	232 ± 18	599 ± 20	1900 ± 50

In ternary nanocomposites, it was observed that by addition of 5 wt.% PANI NPs, the voltage witnessed a remarkable surge. As shown in Fig. 6b, the output voltages in PCL/PANI5/BT20, PCL/PANI5/BT30, and PCL/PANI5/BT40 nanocomposites, surged by approximately 80% to 232 ± 18, 599 ± 20, and 1900 ± 50 mV, respectively (Table 4). PANI NPs had a synergic effect on piezoelectricity and enhanced the output voltage of nanocomposites. Conductive PANI NPs assisted to decrease the internal resistance of ternary nanocomposite by introducing conductive paths^35^.

The conductivity of PCL/PANI/BT nanocomposite mats plays a noticeable role in the resulted voltage and consequently on piezoelectric properties. Electrospun mats are textures consisting of connected and non-connected nanofibers, which are randomly placed in the platform with a large number of porosities. Generated piezoelectric charges in connected nanofibers contributed to output voltage, while charges in non-connected nanofibers remained useless. By inducing the conductivity in nanofibrous mats, the piezoelectric charges can flow through conductive paths to the connected nanofibers’ area and increase the output voltage^36^.

Another possible reason for this change in piezo-response is due to growth in the Young’s modulus and ultimate strain in ternary nanocomposite mats. According to the results in Fig. 5b,c, the PCL/PANI5/BT20, PCL/PANI5/BT30, and PCL/PANI5/BT40 nanocomposites showed an enhancement in Young’s modulus and ultimate tensile stress and strain, compared to binary nanocomposites of PCL/BT with 20, 30, 40 wt.% BT NPs. The piezoelectric voltage of prepared samples under constant tension (2.66 N), is evaluated using the following Eq. (3):

3 $$ V = d_{33} \varepsilon Yt $$

where d_33_ is the piezoelectric constant, ε is for strain, Y is the Young’s modulus and t represents the thickness of the nanofibers, and V is for output voltage. Considering the Eq. (3), By the rise in Y and ε, with constant d33 and t, the output voltage rises accordingly^36^.

### Biocompatibility of nanofibrous scaffolds

**Figure 7:**
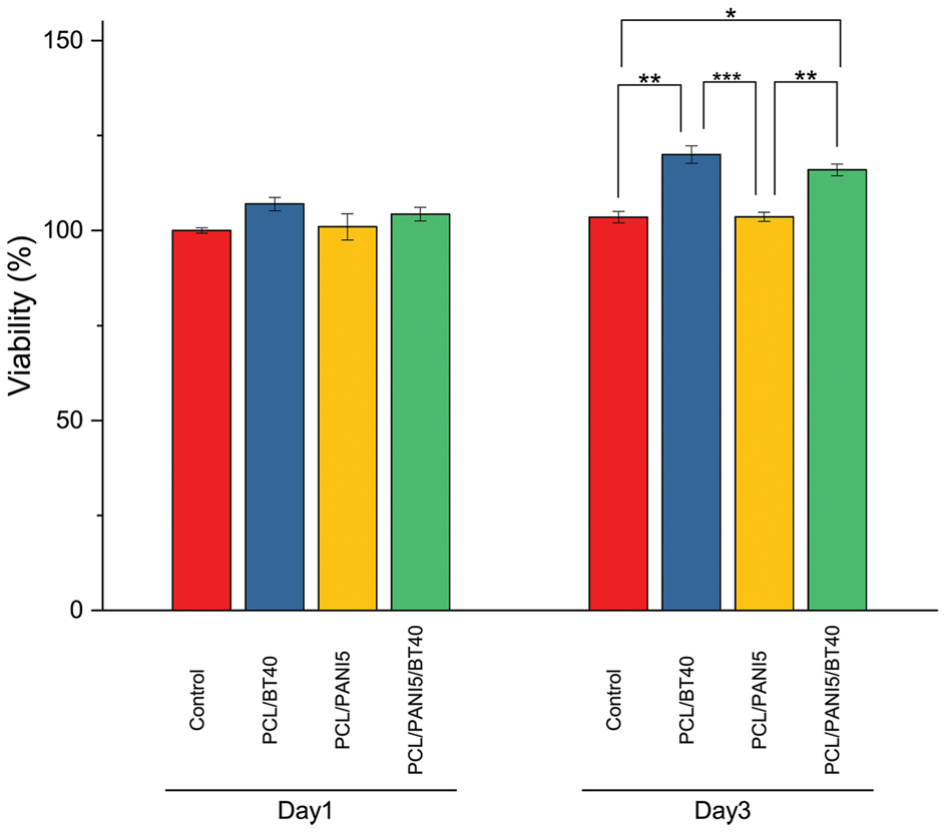
MTT assay, assessment of viability and proliferation of cells on PCL/BT40, PCL/PANI5 and PCL/PANI5/BT40 nanofibers versus times. Results obtained for PCL/PANI5/BT40 sample as a piezo-electrical stimuli conductive nanocomposite showed that the scaffold is a surface-active platform for cells adhesion and growth via enhancing the cell growth and proliferation, where viability reached 104 ± 3% and 116 ± 2% on days 1 and 3, respectively.

MTT assay was conducted to evaluate the biocompatibility of the binary (PCL/BT40 & PCL/PANI5) and ternary (PCL/PANI5/BT40) nanocomposite samples. Cells were cultured beside the samples and their viability and proliferation were checked out on days 1 and 3. The total results indicated that none of the samples showed toxicity and cell growth and proliferation were observed in all samples. However, PCL/PANI5 sample showed the lowest proliferation compared to other samples. This can be due to the presence of PANI NPs and the possibility of their toxicity due to the release of dopant acids^37,38^. As previous studies have been asserted, BT NPs have a positive role in the attachment and proliferation of cells^39,40^. As it can be seen in Fig. 7, in the sample PCL/BT40, a significant increase of up to 120 ± 4% has witnessed in the cell viability on day 3. Results obtained for PCL/PANI5/BT40 sample as a piezo-electrical stimuli conductive nanocomposite showed that it enhanced the cell growth and proliferation where viability reached 104 ± 3% and 116 ± 2% on days 1 and 3, respectively; which means that the scaffold is a surface-active platform for cells adhesion and growth.

## Conclusions

Conductive scaffolds are a topic that has attracted a great deal of research to date. However, creating a conductive scaffold that can generate electrical signals needless of utilizing external power supplies is a novel approach. The piezo-electrical stimuli conductive scaffold is capable to produce electrical pulse by the body’s natural movement and conduct the electric pulse all over the scaffold, simultaneously. In this study, a novel piezo-electrical stimuli scaffold with high electrical conductivity was designed for tissue engineering applications. For this aim, Barium Titanate NPs as a well-known ferroelectric ceramic with superior piezoelectric properties and high biocompatibility were used as a piezoelectric material within the PCL as a biodegradable, biocompatible polymeric carrier. Binary doped high conductive polyaniline was employed to induce conductivity. The electrospinning method was used to fabricate nanofibrous scaffolds. Various samples with different percentages of BT NPs were produced and characterized. SEM results confirmed that BT NPs have been distributed and embedded inside PCL fibers quite appropriately.

Based on our experiments, the ternary nanocomposite PCL/PANI5/BT40 was chosen as the best candidate for tissue engineering applications. The PCL nanofibers containing the PANI and BT NPs were homogeneously intertwined, resulting an average fiber diameter of 288 ± 180 nm. Also, this composite had a contact angle of 92 ± 7°, making it a desirable surface for cell attachment and protein interactions. Moreover, Young’s modulus, ultimate tensile stress, and elongation were reported as 11 ± 1 MPa, 5 ± 0.6 MPa, and 109 ± 15% respectively. Our experiments indicated that PANI NPs had a synergic effect on the BT piezoelectricity and enhanced the output voltage of PCL/PANI5/BT40 nanocomposites to the 1900 ± 50 mV. Furthermore, our results showed that the chosen scaffold enhanced the cell growth and proliferation where viability reached 104 ± 3% and 116 ± 2% on days 1 and 3, respectively; which means that the scaffold is a surface-active platform for cells adhesion and growth. Results of all performed tests proved the potential of prepared piezo-electrical stimuli conductive scaffolds for a range of tissue engineering applications, especially bone, cartilage, cardiac muscles, and nerve regeneration.

## Data Availability

The datasets generated and/or analyzed during the current study are available from the corresponding author on reasonable request.
